# Age as an Overlooked Element of Diversity: Approaches to Addressing Intergenerational Perspectives

**DOI:** 10.1093/geroni/igaa057.1712

**Published:** 2020-12-16

**Authors:** Allyson Graf, Amy Knepple Carney

## Abstract

Outside of gerontology, age is an often underappreciated element of diversity. At a time when all generations must work together to provide inclusive, multi-faceted solutions to today’s societal problems, ageist and generational stereotypes are often barriers to meaningful intergenerational exchanges. Age derogation and negative stereotypes have been used to splinter communities, perpetuate misinformation, and trivialize intergenerational conversations. As researchers, educators, and practitioners, we understand why age matters, but our students, community leaders, and employers may not. It is our disciplinary obligation to convince those who ignore, dismiss, or misrepresent age of the importance of this aspect of diversity for navigating any multigenerational setting. In this talk, we provide three approaches to addressing age-related beliefs in the classroom. We begin by exploring the impact of generational stereotypes within minority communities. For the LGBT community, negative stereotypes coupled with rapid social change have lead to a growing generational gap. We then shift perspectives to examine the role that lifespan developmental psychology can play in preparing students to enter a diverse multigenerational workforce. Here, we discuss research on age identity and generational identity as distinct and self-enhancing life-span processes, and highlight the developmental barriers that must be navigated in order to foster intergenerational cohesion. Finally, we discuss the findings from Generation to Generation, an intergenerational discussion course for older and younger adults, designed to promote productive intergenerational contact. The results provide evidence that intergenerational discussion may facilitate improved connections between generations.

